# A Miniature Fiber Optic Refractive Index Sensor Built in a MEMS-Based Microchannel

**DOI:** 10.3390/s110101078

**Published:** 2011-01-19

**Authors:** Ye Tian, Wenhui Wang, Nan Wu, Xiaotian Zou, Charles Guthy, Xingwei Wang

**Affiliations:** 1 Department of Electrical and Computer Engineering, University of Massachusetts Lowell, One University Ave., Lowell, MA 01854, USA; E-Mails: wwhclp02@gmail.com (W.W.); nan_wu@student.uml.edu (N.W.); Charles_Guthy@student.uml.edu (C.G.); 2 Biomedical Engineering and Biotechnology Doctoral Program, University of Massachusetts Lowell, One University Ave., Lowell, MA 01854, USA; E-Mail: xiaotian_zou@student.uml.edu (X.Z.)

**Keywords:** Fabry-Perot, optical fiber sensor, refractive index, MEMS

## Abstract

A small, highly sensitive, and electromagnetic interference (EMI)-immune refractive index (RI) sensor based on the Fabry-Perot (FP) interferometer is presented. The sensor’s FP cavity was fabricated by aligning two metal-deposited, single-mode optical fiber endfaces inside a microchannel on a silicon chip. The mirrors on the fiber endfaces were made of thermal-deposited metal films, which provided the high finesse necessary to produce a highly sensitive sensor. Microelectromechanical systems (MEMS) fabrication techniques, specifically photolithography and deep dry etching, were used to precisely control the profile and depth of the microchannel on the silicon chip with an accuracy of 2 μm. The RI change within the FP cavity was determined by demodulating the transmission spectrum phase shift. The sensitivity and finesse of the transmission spectrum were controlled by adjusting the cavity length and the thickness of the deposited metal. Our experimental results showed that the sensor’s sensitivity was 665.90 nm/RIU (RI Unit), and the limit of detection was 6 × 10^−6^ RIU. Using MEMS fabrication techniques to fabricate these sensors could make high yield mass production a real possibility. Multiple sensors could be integrated on a single small silicon chip to simultaneously measure RI, temperature, and biomolecule targets.

## 1. Introduction

Optical refractive index (RI) sensors are a very attractive sensing option for a wide variety of environmental, chemical, and biomedical sensing applications because of their high sensitivity, fast response, ease of fabrication, immunity to electromagnetic interference, and survivability in harsh environments. There are many different kinds of optical RI sensors, each with their own benefits and drawbacks. Surface plasma resonance (SPR) RI sensors can offer high resolution, but their dynamic range is very limited [1]. Grating-based RI sensors have high sensitivity and dynamic range, but their fabrication costs prohibit their use in many applications, and the relatively long length of the gratings limit their applications as point sensors [2,3]. Tapered fiber RI sensors’ tiny structures are suitable as point sensors, but their unstable high-order mode interference and extreme sensitivity to different kinds of environmental changes, such as temperature, humidity, and mechanical stress, can negatively affect their measurement accuracy [4–6]. In contrast to all these RI sensors, Fabry-Perot (FP) interferometric RI sensors can provide cost-effective RI measurement [7–9] with a low detection limit, which is critical in biomedical applications [10–12].

Fiber-optic FP interferometric sensors can be categorized into two groups: intrinsic and extrinsic. In intrinsic FP sensors, the sensing cavity is between two dielectric mirrors inside the fiber [13]. In extrinsic FP sensors, the sensing cavity is between two cleaved fiber endfaces that are aligned and bonded within a channel [14]. For both kinds of FP sensors, the reflections at the dielectric mirrors or endfaces of the cavity form an interference pattern whose optical path is determined by the length and RI of the cavity. The phase shift or the fringe contrast change of the interference signal corresponds to the change of the RI [15].

Most intrinsic FP sensors are made by bonding or splicing a section of fiber with mirrors coated on both ends between two fibers. Unfortunately, it is difficult to precisely control the film thickness and flatness. In addition, the thin-film mirrors can be easily destroyed during the bonding/splicing process [16]. Alternatively, femtosecond lasers can be used to fabricate the sensing cavity in intrinsic FP sensors. Due to the low reflectivity of the laser-ablated surface, however, multiple reflections negligibly contribute to the resulting low-finesse interference [17,18]. For extrinsic FP sensors, alignment is a considerable challenge [19], but can be overcome by using precisely etched microchannels. Integration of optical sensing functionalities into a microfluidic device is desirable in the fabrication of a compact, portable, and automated sensor [20].

This paper presents a simple extrinsic FP fiber sensor that measures the RI of a liquid in a silicon microchannel. Using a microchannel structure, the sensor can be integrated into a microfluidic device. The sensor consists of a short air cavity between two chromium/gold-deposited endfaces on a single-mode fiber. A thin chromium (Cr) layer is coated onto the tips of each optical fiber before the gold (Au) is deposited in order to improve the durability of the gold endfaces. The high reflectivity of the two endfaces leads to a high finesse interference of the light transmission spectrum, and the phase shift can be easily detected and correlated to RI variation. A response curve for temperature calibration during RI testing is determined. The sensitivity and finesse of the transmission spectrum can be controlled by adjusting the cavity length and the thickness of the gold films at the endfaces [21]. By using MEMS fabrication techniques, the sensor can be manufactured quickly and inexpensively. Multiple sensors can be integrated in a small chip for simultaneous measurements of RI, temperature, and biomolecule targets.

## 2. Materials and Fabrication Method

**Figure 1.**
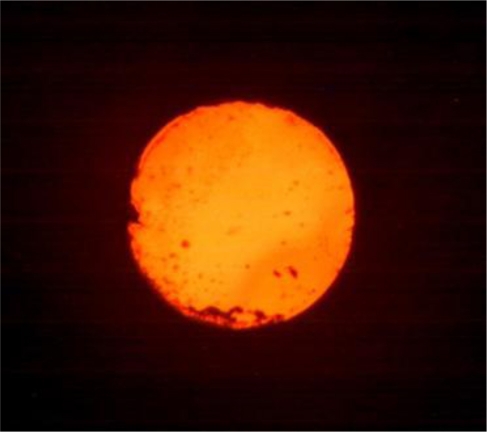
Metal-deposited fiber endface. The center bright area is the 125 μm fiber endface covered by Cr-Au layer. Only some minor erosion around the endface’s edge were observed six months after metal deposition.

Single-mode fibers (SMF-28) with core/cladding diameters of 8/125 μm were used in the fabrication of the sensor. The protective coating was removed from a 5 mm long section after the fiber tip was cleaved. The cleaved angle of the fiber endface was controlled with an accuracy of 0.5 degrees as measured by a fusion splicer (Fitel S177A). A bundle of these fibers were put into a thermal evaporation chamber with their endfaces facing a metal target. Because of its high hardness and resistance to corrosion, a thin chromium layer was then coated onto the endface before gold was deposited. This improved the mirrors’ durability while being heated by a laser [22]. A typical metal-deposited fiber endface is shown in Figure 1.

**Figure 2.**
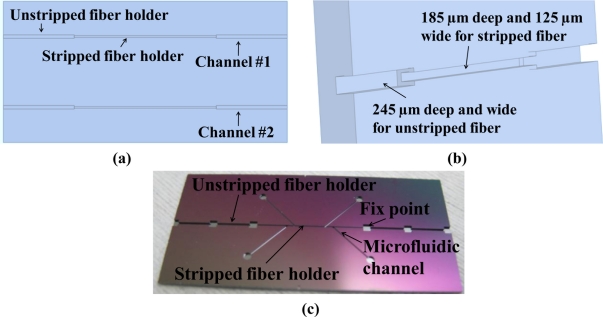
**(a)** Microchannel chip structure. Multiple channels can support multiple sensors on a single small silicon chip to simultaneously measure RI and temperature. The silicon chip can easily be integrated into a micro fluidic system for biosensing applications. **(b)** Microchannel depth difference. Both ends of the channel were 245 μm wide and 245 μm deep so they could hold the unstripped sections of the fiber, while the middle of the channel was 125 μm wide and 185 μm deep so it could hold the stripped fiber. **(c)** Real etched wafer. The rectangular fix points were left for bonding the fibers, and the microfluidic channels were left to further improve the testing control.

The microchannel for fiber alignment was etched onto a silicon wafer with an inductively coupled plasma deep etcher after the wafer was patterned by a layer of positive-tuned photoresist. Two microchannels on a silicon chip are shown in Figure 2(a). As seen in Figure 2(b), both ends of the channel were 245 μm wide and 245 μm deep so they could hold the unstripped sections of the fiber, while the middle of the channel was 125 μm wide and 185 μm deep so it could hold the stripped fiber. The different depths ensured that the gold endface of the 125 μm fiber could be immersed into an RI solution without vibration. All depths and widths were accurate to within 2 μm. The channel sizes were matched to fit the fiber dimensions for correct alignment. This microchannel fabrication method was found to be easier than the traditional method of microchannel fabrication, which produces V-grooves by silicon anisotropic etching.

**Figure 3.**
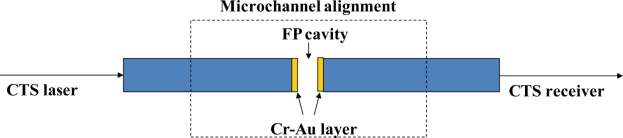
Alignment of the metal-deposited fiber endfaces. A CTS was used to monitor the transmission spectrum response during the alignment process under a Stereo Zoom Binocular Microscope.

A laser (scan wavelength 1,520 nm to 1,570 nm) in a component test system (CTS) (Si 720, Micron Optics) excited one end of the fiber, while the spectrum response from the other side of the fiber was monitored. The alignment was monitored using a Stereo Zoom Binocular Microscope (Scienscope NZ Series). Figure 3 shows the schematic of the fiber alignment and CTS test.

Two six-dimensional stages were used to finely position the two fiber endfaces within the microchannel to ensure optimal alignment. The FP cavity length was also controlled using the stages. When the desired cavity length and transmission spectrum were achieved, epoxy with a low coefficient of thermal expansion was applied to fix the two fibers’ positions inside the microchannel. An epoxy-free packaging method, such as thermal bonding, is under development for applications with large temperature variations, and is expected to help the sensor achieve better thermal stability.

## 3. Results and Discussion

### 3.1. Sensor Spectrum

**Figure 4.**
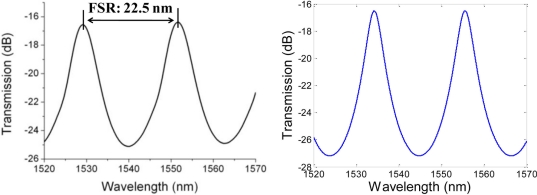
**(a)** Actual transmission spectrum of a sensor with 9 dB contrast and 22.5 nm FSR by experiment. **(b)** Simulated transmission spectrum.

Figure 4(a) shows the transmission spectrum of a typical sensor with an air cavity. The transmission contrast range was between −16 dB and −25 dB, the fringe peak was sharper than the valley, and its free spectral range (FSR) was 22.5 nm. The cavity length *L* can be calculated using the equation:

(1) $$L=\frac{\lambda^{2}}{2\mathrm{nFSR}}$$

where *λ* is the center wavelength (1,552 nm), and *n* is the RI of the air. The calculated cavity length *L* was 53.5 μm, which agreed with our measurement using the microscope.

The value obtained for the cavity length was then used to simulate the spectrum with the equation:

(2) $$I={\left|\frac{{\left(1-r\right)}^{2}}{1-r^{2}e^{i2\frac{2\pi }{\lambda }\mathrm{nL}}}\right|}^{2}$$

where *r* is the endface reflectivity of 0.74 and *n* is again the RI of air. Note that the loss caused by fiber numerical aperture was ignored due to the short cavity length. By scanning the wavelength *λ* from 1,520 nm to 1,570 nm, the results of the MATLAB simulation as seen in Figure 4(b) closely matched the real spectrum. The peak wavelength shift between the spectra of experiment and simulation was caused by the slight difference of the initial cavity length and RI. The simulated spectrum was further used to evaluate the phase shift caused by the RI change. Based on the results of the simulation, it was found that a higher reflectivity obtained by a thicker gold layer on the endface could improve the finesse and contrast of the sensor’s spectrum.

### 3.2. Refractive Index Test

**Figure 5.**
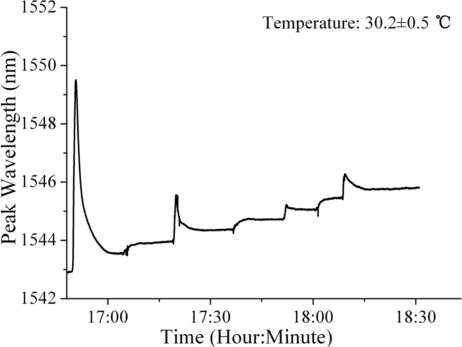
Changes in peak wavelength for a typical trial. Seven sugar samples were added to the solution (RI increased linearly by 6 × 10^−4^ RIU every time), causing a total redshift of 2.8 nm in spectrum, signifying a 4.2 × 10^−3^ RI increase.

Two sensors were separately tested in 300 g of water at 30.2 ± 0.5 °C. The water’s RI was changed by adding a 1 g sample of table sugar, increasing the RI by 6 × 10^−4^ RIU each time. Since the solution was not oversaturated with sugar, its RI increased linearly within the test range under a constant temperature. Over the course of a typical trial, seven 1 g sugar samples were added to the solution. Since the results of every experimental trial for all two sensors were similar, the results of a single trial were shown in Figure 5. The peak wavelengths were found to correspond to the RI changes. When the first and third sugar samples were added to the solution, the RI solution was uneven, causing sudden redshifts in the peak wavelength. After about five to fifteen minutes, the peak wavelength was stable. There was a total redshift of 2.8 nm, signifying a 4.2 × 10^−3^ RI increase.

**Figure 6.**
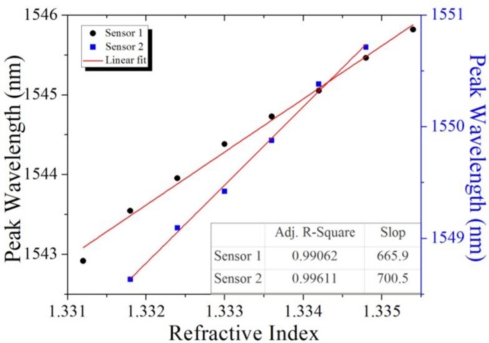
Linearity and sensitivity of the sensors.

As seen in Figure 6 (Sensor 1), the wavelength shift corresponded to the RI increase (after conversion from sugar solution), and demonstrated the sensor’s sensitivity and linearity. The linearity had a correlation coefficient (R^2^) of 0.99062, and its sensitivity was 665.90 nm/RIU using a linear fit. The linearity and sensitivity of another sensor (Sensor 2) were also provided.

**Figure 7.**
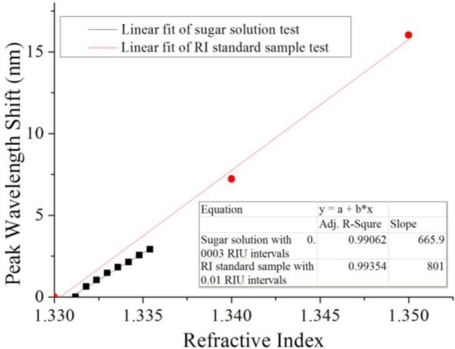
Comparison between sugar solution and RI standard sample test (Sensor 1). The sensor could detect large and small changes in RI equally well.

For a more accurate control of the RI change, RI Liquids (Cargille AAA18032) were tested as standard samples using the same sensor (Sensor 1) to compare the sugar solution test results. As shown in Figure 7, the correlation coefficient of the standard sample measurement was 0.99354, compared to 0.99062 for the sugar solution test results. The small difference in the correlation coefficients meant that the sensor was consistently linear. The measured sensitivities were 801.0 nm/RIU and 665.9 nm/RIU for the RI Liquids and the sugar solution, respectively. The 16% difference in the sensitivities might have been caused by the low precision of the RI increase in the sugar solution. Repeatable linear fits and sensitivities were obtained from two tests of the same sensor. The results proved that the sensor could detect large and small changes in RI equally well.

### 3.3. Temperature Effect

**Figure 8.**
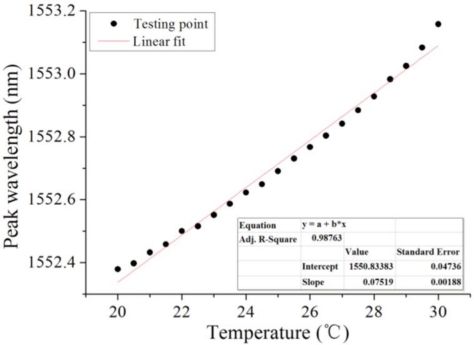
Temperature dependence curve that can be used as a reference to calibrate the real measurements.

The RI test was performed at room temperature, which varied slightly over the course of the test. Our sensor was found to be able to provide accurate measurement in a 30.2 ± 0.5 °C environment. Despite the results of the stable temperature test, ambient temperature still needed to be taken into account, since the thermal expansion of the optical fiber, silicon chip, and bonding adhesive could lead to a spectrum phase shift. To that end, a temperature dependence experiment was designed to evaluate the effect of temperature on measurement. Sensor 1 was placed inside a temperature- and humidity-controlled chamber, where the temperature was ramped up from 20 °C and 30 °C, and the relative humidity was kept at 25%RH. The temperature and peak wavelength shifts were recorded simultaneously. As seen in Figure 8, the temperature sensitivity of the RI sensor was found to be 0.075 nm/°C, based on the linear fit of the experiment data. A dependence curve was generated, which could be used as a reference to calibrate the real measurements.

### 3.4. Discussion

Eliminating the temperature cross-sensitivity was important to improve the sensor’s stability. One of the critical sources of sensor instability was the thermal expansion of the bonding adhesive. To eliminate this source of instability, laser thermal bonding, instead of the bonding adhesive, could be used. The MEMS-based silicon chip was convenient for thermal bonding. The silicon chip could be further processed by plasma-enhanced chemical vapor deposition (PECVD) for a silicon-dioxide layer 2 μm thick which can easily be bonded with the fiber by laser heating. A CO_2_ laser engraving system (Zing - Model 10000) that provides adjustable laser power, speed, and pulse frequency will be used for heating. The next source of sensor instability was the thermal expansion of the silicon wafer. At 25 °C, the coefficient of thermal expansion (CTE) of silicon is 2.6 μm·m^−1^·K^−1^. The total expansion added to the cavity length can be determined by multiplying the thermal coefficient by the distance between the two thermal bonding points. Therefore, by reducing the bonding length L from 10 mm to 1 mm, the temperature cross-sensitivity would be decreased by an order of ten. The thermal expansion of optical fiber was also a negligible source of instability. Fused silica substrate or glass substrate with similar CTEs should be good alternatives for silicon substrate as the microchannel chip. A temperature cross-sensitivity of almost zero could be achieved because of the similar thermal expansions of the optical fibers and the substrate.

Our methodology for measuring the RI was to track the position of the spectral extremes. If infinitely high spectral resolution were available and absolutely zero system noise were present, the sensor performance would be characterized only by its sensitivity. However, neither of those conditions exists in the real world, thus making it difficult to locate the true center of the extreme. As a result, the limit of detection had to be ascertained to determine the finite sensor resolution. From our current results of the spectral noise and the CTS’s resolution, the sensor’s limit of detection was found to be 6 × 10^−6^ RIU. The noise caused by amplitude variations and spectral variations were themselves caused by the device’s resolution and the sensor’s spectral quality. Using a high resolution spectrometer would go a long way in improving the limit of detection. Finesse was another key sensor aspect that can make the real results approach to those obtained during simulation. Better finesse can be achieved through thicker metal deposition on the endfaces.

## 4. Conclusions

A highly-sensitive FP RI sensor was fabricated by aligning and bonding two metal-deposited optical single-mode fiber endfaces within a microchannel on a silicon wafer. A temperature dependence curve was created to calibrate the thermal expansion cross-sensitivity. MEMS fabrication techniques could make high yield manufacturing of these sensors possible. Further, these sensors could be integrated into small silicon chips that could simultaneously detect changes in RI, temperature, and biomolecule targets.
